# The Mantel-Haenszel Procedure Revisited: Models and Generalizations

**DOI:** 10.1371/journal.pone.0058327

**Published:** 2013-03-13

**Authors:** Vaclav Fidler, Nico Nagelkerke

**Affiliations:** 1 Department of Epidemiology, University of Groningen, University Medical Center Groningen, the Netherlands; 2 Institute of Public Health, United Arab Emirates University, UAE; 3 Department of Medical Microbiology and Infectious Diseases, University of Manitoba, Winnipeg, Canada; 4 Department of Public Health, Erasmus Medical Center, Rotterdam, the Netherlands; Université Catholique de Louvain, Belgium

## Abstract

Several statistical methods have been developed for adjusting the Odds Ratio of the relation between two dichotomous variables X and Y for some confounders Z. With the exception of the Mantel-Haenszel method, commonly used methods, notably binary logistic regression, are not symmetrical in X and Y. The classical Mantel-Haenszel method however only works for confounders with a limited number of discrete strata, which limits its utility, and appears to have no basis in statistical models. Here we revisit the Mantel-Haenszel method and propose an extension to continuous and vector valued Z. The idea is to replace the observed cell entries in strata of the Mantel-Haenszel procedure by subject specific classification probabilities for the four possible values of (X,Y) predicted by a suitable statistical model. For situations where X and Y can be treated symmetrically we propose and explore the multinomial logistic model. Under the homogeneity hypothesis, which states that the odds ratio does not depend on Z, the logarithm of the odds ratio estimator can be expressed as a simple linear combination of three parameters of this model. Methods for testing the homogeneity hypothesis are proposed. The relationship between this method and binary logistic regression is explored. A numerical example using survey data is presented.

## Introduction

The practice of exploring residual association between two variables X and Y after adjusting for other, confounding, variables Z is at the heart of much of statistical and epidemiological analysis. It underlies the search for potentially causal relationships in observational research. For continuous X and Y the partial correlation coefficient is the most widely used measure of adjusted association and presents the correlation between X and Y if Z would be kept fixed (constant). The (partial) regression coefficients, of either the regression of Y on X and Z or the regression of X on Y and Z are also measures of association between X and Y that are adjusted for Z, but these measures are not symmetrical in X and Y. Such asymmetrical measures are sometimes adequate, especially when one of the two variables X and Y is obviously the dependent and the other the independent variable, e.g. when a causal relationship between X and Y exists or is assumed, as is often the case in randomized clinical trials and in observational cohort or case-control studies. In contrast, the partial correlation coefficient is symmetrical in X and Y and is therefore a more logical choice when there is no *a-priori* plausible unidirectional causal link between X and Y, for example when X and Y are the diastolic and systolic blood pressure respectively and Z is age (say), measured in a cross-sectional random population sample.

For dichotomous X and Y that assume only the values 0 and 1 (e.g. alive and dead, or smoker and non-smoker), a commonly used measure of association is the odds ratio 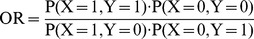



The population OR can not only be estimated from a random population sample, such as a cross-sectional survey, but also from samples stratified with respect to either X or Y, such as a cohort or case-control study. Several methods have been developed for adjusting the association between X and Y for a third variable Z. The best known are the Mantel-Haenszel (MH) method [1], which is symmetrical in X and Y, and logistic regression, which is not [2]. Even in the absence of a direct causal link between X and Y, regressing Y on X and Z generally yields a different estimate (and standard error) of OR(X,Y|Z) than regressing X on Y and Z although the difference is often modest. Differences may arise, for example, when either Z explains more (or less) variation in Y than in X or when there are specification errors in the regression of Y on X and Z or X on Y and Z. Such misspecification can occur, for example, when the true relationship between Y and X and Z is not the logistic model, but (say) a probit model. This lack of symmetry can make logistic regression in this context undesirable. If we want to present, for example, the residual relationship between two cardiovascular risk factors or disorders, with no direct causal link between the two but both potentially influenced by common factors such as gender, then the MH-method would seem a more attractive choice than logistic regression. Its symmetry, as well as its intuitive appeal, i.e. the fact that the procedure can easily be understood without advanced mathematical training, probably explains the enormous popularity of the procedure among epidemiologists and other empirical researchers.

The usual form of writing the MH odds ratio estimate is
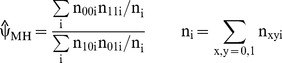
(1)where n_xyi_ denotes the number of observations in a (x,y)-cell of the 2-by-2 table for the i–th stratum and where the summation is over all strata of Z. The method has only been developed for Z with a limited number of levels of exact matches (the ‘strata’), which is the case when Z is a single categorical variable, such as sex, or when strata were created by design, e.g. by matching. This is because in calculating the MH odds ratio all observations at stratum Z for which any of the marginal totals of the X_Z_-by-Y_Z_ table are zero are ignored. Thus if combinations of Z are unique for each subject then all observations are ignored!

Attempts to fix this shortcoming, such as Miettinen's multivariate confounder (discriminant) score method, which has poor statistical properties [3], [4] and seems to be forgotten, were not successful. Yet another approach is that of using binary logistic models for marginal probabilities P(Y = 1|Z) and P(X = 1|Z), and then expressing P(X = 1,Y = 1|Z) as a function of these marginal probabilities and of the odds ratio, and maximizing the likelihood function with respect to the odds ratio and the parameters of marginal distributions. This approach has been explored by Carey *et al* [5] and le Cessie and van Houwelingen [6]. It requires special software to fit the models and is not equivalent to the Mantel-Haenszel method when Z is a one-dimensional categorical variable.

We here propose a very simple method to extend the MH odds ratio to a general case of Z being an m-dimensional vector of covariates, some of which may be continuous. Its basic idea is to replace Mantel-Haenszel cell entries with subject-specific classification probabilities generated by a suitable multinomial model. As presenting an adjusted OR as a summary measure of association makes primarily sense if subject-specific odds ratios can be assumed not to depend on Z, i.e. under the hypothesis of homogeneity of the OR across subjects (strata, levels of Z), we also address estimation of the OR under the assumption of homogeneity and discuss how to test this homogeneity.

## Methods and Results

### Extended Mantel-Haenszel odds ratio estimate

If the subjects form strata S_i_ of size n_i_ and if p_xyi_′s denote the observed fractions (probabilities) in each stratum, p_xyi_ = n_xyi_/n_i_, then
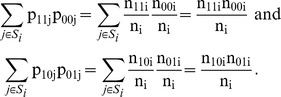



The expression (1) can then be written in terms of observed probabilities as 
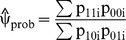
(2)where the sum is over all subjects. This probabilistic formulation suggests a generalization of (2) in which p_xyi_ denotes an estimated probability P(X = x,Y = y|Z = z_i_) for the i-th subject with (possibly vector-) covariate z_i_ (and where the sum is over all subjects).

The estimates p_xyi_ can be obtained from any suitable regression model. A convenient and widely used model is the multinomial logistic regression model
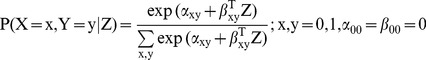
(3)with 3 intercept parameters α and 3•m parameters β_xy_ = (β_xy1_,…, β_xym_)^T^, where m is the dimension of the covariate vector Z = (Z_1_,…,Z_m_)^T^. This model has strong connections to other important statistical models, specifically the log-linear model [7]. Classification probabilities p_xyi_ can be obtained from (3) using maximum likelihood (ML) estimates of α_xy_ and β_xy_ obtained with standard software, such as SPSS (nomreg), STATA (mlogit), R (library nnet) and SAS (proc logistic), and the OR estimate 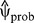
can be readily computed using (2). Note that 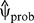 can be also interpreted as a weighted mean of subject specific OR estimates (p_11i_ p_00i_)/(p_10i_ p_01i_). The standard error (SE) of 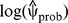 is derived in Appendix S1 and can be used to calculate 95% confidence intervals for the OR by exponentiating the two confidence limits 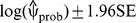
for the 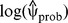.

### The odds ratio as a model parameter in the multinomial logistic model

The subject-specific log odds ratio 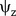
under the multinomial logistic model (3) is
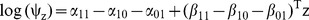
(4a)


This suggests an alternative estimator 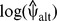 of the log(OR) as the average of subject-specific quantities 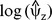
computed directly from the ML-parameter estimates using (4a). The subject-specific odds ratio 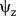 generally depends on Z unless δ = β_11_-β_01_-β_10_ equals zero, which presumably defines the situation where a ‘summary’ OR is most meaningful. Testing of the hypothesis H_0_: δ  = 0of homogeneity of odds ratios can be carried out by the Wald test or by the likelihood ratio (LR) test. To carry out the LR-test and to obtain ML-estimates under the constrained model, i.e. under H_0_: δ  = 0, we do need to fit this model. This produces the ML-estimate of log(ψ),
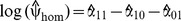
(4b)and of its standard error. This ML-estimate is identical to the Mantel-Haenszel type estimate (2) computed from classifications probabilities derived from the homogeneity model:
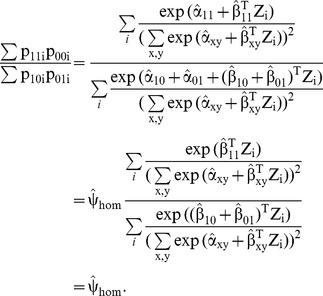



This demonstrates the close link between the classical MH-approach and our model based OR estimate. Computations can be carried out in R [8] using the package partialOR [9]; Appendix S2 gives an example.

### The odds ratio in binary logistic regression and its relationship to the multinomial logistic model

To explore the relationship between the multinomial logistic model and the two binary logistic regression models (Y on X, Z and X on Y, Z) commonly used to adjust the OR between X and Y we note that from the multinomial logistic model (3) we can derive these two versions of binary logistic regression models, as follows:
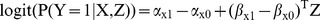
(5a)

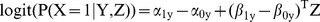
(5b)


The model (5a) can rewritten as
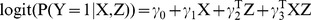
where γ_0_ = α_01_−α_00_ = α_01_, γ_1_ = α_11_+α_00_−α_10_−α_01_ = α_11_−α_10_−α_01_, γ_2_ = β_01_−β_00_ =  β_01_, γ_3_ = β_11_+β_00_−β_10_−β_01_ = β_11_−β_10_−β_01_. For model (5b) we obtain a similar expression. To fit model (5a) to data we enter X, Z and the interaction term X•Z in the model, and similarly for (5b). Homogeneity of OR′s under the multinomial logistic model with δ = 0 is equivalent to absence of interaction (γ_3_ = 0) under the logistic model (5a), i.e. with Z being only a confounder and not also an effect-modifier. Under this model log(ψ) = γ_1_ is the same parameter as that estimated under the multinomial logistic model (3) with δ = 0. The ML-estimates of ψ may however differ (albeit not much) as the likelihood functions differ. Note that model (3) can be either factorized as P(Y|X,Z)•P(X|Z) or as P(X|Y,Z)•P(Y|Z). When fitting models (5a) or (5b) we ignore the marginal distributions of X given Z or of Y given Z, respectively, which are implicitly modeled in (3). Also if δ ≠ 0 and – as is usually done – the interaction is ignored in the logistic regressions then the two logistic regression models are misspecified and the adjusted OR estimates are likely to differ as well. Assuming absence of interactions and model misspecifications models (5a) and (5b) simplify to α_01_+log(ψ)X+ β_01_^T^Z and α_10_+log(ψ)Y+ β_10_^T^Z, respectively, demonstrating that, under these conditions, these two logistic regressions estimate essentially the same parameter log(ψ).

### Model choice

Which of the three models to use: (3), (5a) or (5b)? The assumed design – a random population sample – suggests the multinomial logistic model (3). It leads to an intrinsically symmetrical OR estimate 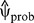 (or, alternatively, 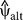). For a more refined analysis we would fit model (3) and carry out a formal test of homogeneity, and if justified by apparent homogeneity use the ML-estimate (4b). In case of heterogeneity we would use either the predicted probabilities p_xyi_ to calculate OR for each subject, or subject specific log(OR) values given by 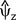, and use them to explore their relation to covariates Z in more detail.

### Example

We used the proposed methods to explore the relationship between (ever) smoking and antibodies (lifelong after infection) to the sexually transmitted viral infection HSV-2 (persists lifelong). For this, USA National Health and Nutrition Examination Survey (NHANES) data were obtained [10]. (NHANES is conducted to assess the health and nutritional status of adults and children in the United States.) Both variables are probably associated with (measured) sexual risk behavior, gender, ethnicity etc. which may thus act as confounders in their relationship. However, there may also be other relationships, e.g. both smoking and HSV-2 infection may be influenced by the (unmeasured) type of social/sexual networks that individuals take part in, giving rise to residual confounding. After elimination of cases with missing and improbable values (e.g. reported first sexual contact at age 1), and subjects reporting never to have had sexual relationships, we obtained a dataset of 991 women and 765 men with complete data. NHANES sampling weights were ignored for this example. The unadjusted OR of the relationship between smoking and HSV-2 was 1.715 (95% CI 1.372-2.144). We were interested in the residual OR after adjustment for age, age at first sexual contact, African American ethnicity, gender, and reported number of lifetime partners (grouped into 1–4, 5–14, 15–39, 40+). Logistic regression with HSV-2 as the dependent variable, yielded an adjusted OR of 1.538 (95% CI 1.176–2.012), and logistic regression with smoking as the dependent variable an adjusted OR of 1.589 (95% CI 1.217–2.075); the closeness of these two LR estimates appears to be consistent with (approximate) homogeneity of the OR. The MH-type OR 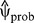
calculated using (2), i.e. the unconstrained symmetrical OR estimate, was 1.550 (95% CI: 1.183-2.022), see Appendix S2. The likelihood ratio test (df = 7) of the constancy of OR's gave a P-value 0.46, thus suggesting that the odds ratio does not depend on the covariates. Therefore, using the parametric method (4b) with δ = 0 was considered appropriate, which yielded an OR estimate 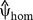
of 1.582 (95% CI: 1.212–2.065). The estimate proposed by le Cessie and van Houwelingen was also close, *viz.* 1.553 (95% CI 1.183–2.032). These adjusted OR values all suggest that the association between smoking and HSV-2 infection is only partially accounted for by association with the above mentioned covariates.

## Discussion

We proposed a method to adjust an Odds Ratio between two dichotomous variables X and Y for other, ‘confounding’, variables Z, that is symmetrical in X and Y. The basic idea is to replace the observed cell entries in strata of the Mantel-Haenszel procedure by estimated classification probabilities estimated from a statistical model, for which we specifically propose and explore the multinomial logistic regression model. In the case of a simple categorical Z the proposed OR estimator is identical to the classical Mantel-Haenszel estimator.

One of the strengths of the multinomial logistic model is that the OR can also be estimated directly from the model parameter estimates. In the important case of homogeneity, that is when the subject specific ORs are independent of Z and thus all identical, the log(OR) estimator simplifies to a simple linear combination of 3 model parameters. We propose the latter estimator as a suitable symmetrical adjusted OR estimate and recommend its use for all situations where a symmetrical adjusted OR is called for. We note that care is needed when applying these methods: an adjustment for variables that appear to be confounders, but are not, may lead to misleading conclusions about the true, causal, associations between variables [11], [12]. Future research could address goodness-of-fit of the multinomial logistic regression model in this context and alternatives, or generalizations, to this model for situations where it is misspecified.

## Supporting Information

Appendix S1 **Calculating the variance of the logarithm of the model-based generalized MH odds ratio, using the delta method.**
(PDF)

Appendix S2 **Example of software code in R.**
(PDF)
