# Supplementary material for: The Mantel-Haenszel Procedure Revisited: Models and Generalizations
Source: PLoS One. 2013 Mar 13;8(3):e58327. doi: 10.1371/journal.pone.0058327 (PMC3596394; doi:10.1371/journal.pone.0058327)
Supplement: Appendix S1 — Calculating the variance of the logarithm of the model-based generalized MH odds ratio, using the delta method. (PDF) [file pone.0058327.s001.pdf]

APPENDIX S1

Calculating the variance of the logarithm of the model-based generalized MH odds ratio, using the delta method

It is easiest to develop our expressions in terms of the extended Z_i vector with the first element =1; similarly the first elements of the β_{xy} vectors are thus = α_{xy}. The parameter estimates are denoted by b_{xy}. According to (2) we have

$$\log(OR) = \log(\hat{\psi}_{prob}) = \log\{\Sigma\, p_{00i}\, p_{11i}\} - \log\{\Sigma\, p_{01i}\, p_{10i}\}$$

where $p_{xyi} = \exp(b_{xy}{}^T Z_i) / \Sigma\exp(b_{uv}{}^T Z_i)$ for x,y=0,1. Recall that b_{00} is a null vector.

The delta method to calculate the variance gives:

$$Var(\log(OR)) = (d(\log(OR)/db))^T \mathbf{V}(d\log(OR)/db)$$

where **V** is the covariance matrix of the parameters (estimated by multinomial logistic regression), and (d(log(OR))/db) is the vector of derivatives of log(OR) with respect to the parameters $\beta = (\beta_{01}, \beta_{10}, \beta_{11})^T$ at point $b = (b_{01}, b_{10}, b_{11})^T$.

These derivatives are as follows:

$$d\log(OR)/db = \{\Sigma\, p_{00i}\, p_{11i}\}^{-1}\, \Sigma(p_{i00}\, dp_{11i}/db + p_{11i}\, dp_{00i}/db)$$
$$- \{\Sigma\, p_{01i}\, p_{10i}\}^{-1}\, \Sigma(p_{01i}\, dp_{10i}/db + p_{10i}\, dp_{01i}/db)$$

$$d\{p_{00i}, p_{01i}, p_{10i}, p_{11i}\}/db_{01} = p_{01i}\{ -p_{00i},\ (1- p_{i01}),\ -p_{i10},\ -p_{i11}\}\, Z_i$$

$$d\{p_{00i}, p_{01i}, p_{10i}, p_{11i}\}/db_{10} = p_{10i}\{ -p_{00i},\ -p_{01i},\ (1-p_{10i}),\ -p_{11i}\}\, Z_i$$

$$d\{p_{00i}, p_{01i}, p_{10i}, p_{11i}\}/db_{11} = p_{11i}\{ -p_{00i},\ -p_{01i},\ -p_{10i},\ (1-p_{11i})\}\, Z_i$$

so that

$$d\log(OR)/db_{01} = \{\Sigma\, p_{00i}\, p_{11i}\}^{-1}\, \Sigma(-2)p_{00i}\, p_{01i}\, p_{11i}\, Z_i - \{\Sigma\, p_{01i}\, p_{10i}\}^{-1}\, \Sigma p_{01i}\, p_{10i}(1-2\, p_{01i})\, Z_i$$

$$d\log(OR)/db_{10} = \{\Sigma\, p_{00i}\, p_{11i}\}^{-1}\, \Sigma(-2)p_{00i}\, p_{10i}\, p_{11i}\, Z_i - \{\Sigma\, p_{01i}\, p_{10i}\}^{-1}\, \Sigma p_{01i}\, p_{10i}(1-2\, p_{10i})\, Z_i$$

$$d\log(OR)/db_{11} = \{\Sigma\, p_{00i}\, p_{11i}\}^{-1}\, \Sigma p_{00i}\, p_{11i}\, (1-2p_{11i})\, Z_i + \{\Sigma\, p_{01i}\, p_{10i}\}^{-1}\, 2\Sigma p_{01i}\, p_{10i}\, p_{11i}\, Z_i\ .$$
