# Supplementary material for: The Mantel-Haenszel Procedure Revisited: Models and Generalizations
Source: PLoS One. 2013 Mar 13;8(3):e58327. doi: 10.1371/journal.pone.0058327 (PMC3596394; doi:10.1371/journal.pone.0058327)
Supplement: Appendix S2 — Example of software code in R. (PDF) [file pone.0058327.s002.pdf]

APPENDIX S2

Example of software code in R

Data used in our example were prepared in R in the form of a data frame `dataNHANES` with binary variables x (smoking) and y (HIV-2 status) and with 7 explanatory variables. These were: dummy-variables pn2, pn3, pn4 representing the four categories of lifetime partners, two binary variables ethn (African American ethnicity) and gender, and two continuous variables, age and age1 (age at first sex). It is assumed that the package partialOR [9] is installed in R. To perform the calculations the following commands are given at R prompt:

```
> library(partialOR)
> partialOR(dataNHANES)
```

The output starts with several lines with information on convergence of the optimalization routine (not shown here). It continues with:

```
Partial Odds Ratio estimation
Model        Deviance
Null          4289.45
Full          3677.41
Homogen.      3684.10
LR-test of homogeneity: LR = 6.697 , df = 7 , P-value = 0.4611
 Unadjusted OR:
   log(OR) = 0.5395 , SE = 0.1138
   OR = 1.715 ,  95%-CI:  1.372 to 2.144
 MH-type Full model estimate:
   log(OR) = 0.438 , SE = 0.1358
   OR = 1.550 ,  95%-CI:  1.187 to 2.022
 Homogeneity-model estimate:
   log(OR) = 0.4585 , SE = 0.136
   OR = 1.582 ,  95%-CI:  1.211 to  2.065
```

The Null model is the multinomial logistic model with 3 intercept parameters. The Full model uses 3+3x7=24 parameters, the Homogeneity model 3+2x7=17 parameters. To obtain detailed information we would use the function `fitOR()`. This function returns a list object with the estimated multinomial logistic model parameters and other quantities.
